# Accelerometer-derived physical activity and sedentary time by cancer type in the United States

**DOI:** 10.1371/journal.pone.0182554

**Published:** 2017-08-14

**Authors:** Keith M. Thraen-Borowski, Keith P. Gennuso, Lisa Cadmus-Bertram

**Affiliations:** 1 Department of Kinesiology, Loras College, Dubuque, Iowa, United States of America; 2 Department of Biology, Loras College, Dubuque, Iowa, United States of America; 3 Department of Kinesiology, University of Wisconsin-Madison, Madison, Wisconsin, United States of America; 4 University of Wisconsin Population Health Institute, University of Wisconsin-Madison, Madison, Wisconsin, United States of America; 5 Paul P. Carbone Comprehensive Cancer Center, University of Wisconsin-Madison, Madison, Wisconsin, United States of America; Indiana University, UNITED STATES

## Abstract

The 2003–2004 and 2005–2006 cycles of the National Health and Nutrition Examination Survey (NHANES) were among the first population-level studies to incorporate objectively measured physical activity and sedentary behavior, allowing for greater understanding of these behaviors. However, there has yet to be a comprehensive examination of these data in cancer survivors, including short- and long-term survivors of all cancer types. Therefore, the purpose of this analysis was to use these data to describe activity behaviors in short- and long-term cancer survivors of various types. A secondary aim was to compare activity patterns of cancer survivors to that of the general population. Cancer survivors (n = 508) and age-matched individuals not diagnosed with cancer (n = 1,016) were identified from a subsample of adults with activity measured by accelerometer. Physical activity and sedentary behavior were summarized across cancer type and demographics; multivariate regression was used to evaluate differences between survivors and those not diagnosed with cancer. On average, cancer survivors were 61.4 (95% CI: 59.6, 63.2) years of age; 57% were female. Physical activity and sedentary behavior patterns varied by cancer diagnosis, demographic variables, and time since diagnosis. Survivors performed 307 min/day of light-intensity physical activity (95% CI: 295, 319), 16 min/day of moderate-vigorous intensity activity (95% CI: 14, 17); only 8% met physical activity recommendations. These individuals also reported 519 (CI: 506, 532) minutes of sedentary time, with 86 (CI: 84, 88) breaks in sedentary behavior per day. Compared to non-cancer survivors, after adjustment for potential confounders, survivors performed less light-intensity activity (P = 0.01), were more sedentary (*P* = 0.01), and took fewer breaks in sedentary time (*P* = 0.04), though there were no differences in any other activity variables. These results suggest that cancer survivors are insufficiently active. Relative to adults of similar age not diagnosed with cancer, they engage in more sedentary time with fewer breaks. As such, sedentary behavior and light-intensity activity may be important intervention targets, particularly for those for whom moderate-to-vigorous activity is not well accepted.

## Introduction

In the last few decades, much work has been dedicated to the study of physical activity in cancer survivorship [1–4]. Collectively, this research has suggested that physical activity is safe both during and after cancer treatment [4] and may result in improvements in physical functioning, quality of life, and, in certain cancer types, cancer-related fatigue [5–7]. Within the past decade, sedentary behavior has gained increased attention as a health behavior distinct from physical inactivity and as a potential independent predictor of health outcomes [8–11]. Sedentary behavior includes any waking activity that is performed while sitting or reclining that does not substantially increase energy expenditure [12]. Prolonged sedentary behavior may be associated with increased prostate, colorectal, endometrial, and ovarian cancer risk, cancer mortality in women, as well as weight gain and BMI in breast and colorectal cancer survivors [13,14]. The putative mechanisms underlying these associations include the potential for excess sedentary time to promote weight gain and obesity, insulin dysregulation, and low-grade chronic inflammation [13].

Current understanding of the relationship between physical activity, sedentary behavior, and cancer survivorship may be limited by the lack of objective measurement of these behaviors in many studies. Physical activity and sedentary behavior are most commonly assessed using self-report questionnaires [15–17], which are prone to recall error and social desirability bias–including over-reporting of moderate- and vigorous-intensity physical activity and underestimating sedentary behavior [18]. They also tend to be especially poor at capturing routine, non-repetitive daily activities, such as non-exercise light-intensity physical activity (*e*.*g*., light activity including housework and other daily activities) and sedentary behavior [15,19]–the most frequent types of activity for the general public. These activities, performed dozens of times a day at various bout lengths, are the most difficult to recall and, thus, easiest to misreport. As such, our collective understanding of participation in physical activity and sedentary behavior in the cancer survivorship population is limited. One way to address the limitations of self-report measures is the use of an objective measure of physical activity and sedentary behavior, such as accelerometry. Accelerometers record the total volume of physical activity and sedentary behavior of the wearer, including specifics on duration, intensity, and frequency, while limiting the error and inherent biases often associated with subjective measures [20,21]. The 2003–2004 and 2005–2006 cycles of the National Health and Nutrition Examination Survey (NHANES), a program of cross-sectional studies conducted since 1999 by the National Center for Health Statistics, were among the first population-level studies to incorporate objectively measured physical activity and sedentary behavior via accelerometry, allowing for greater understanding of these behaviors in the U.S. [22]. In a seminal paper using these data, Troiano et al [22] found that the percentage of U.S. adults accumulating at least 30 minutes of moderate-intensity physical activity, the American College of Sports Medicine’s recommended amount of physical activity at the time, was a great deal less than previous work using self-report measures (approximately 5% as compared to 45% [23] - 51% [22]), suggesting that initial estimates of physical activity at the population level were potentially quite overstated.

In the context of cancer survivorship, previous analyses of NHANES accelerometer data have examined physical activity in long-term cancer survivors [24,25] and reported both physical activity and sedentary behavior in breast [26,27] and long-term prostate cancer survivors [28]. However there has yet to be a robust, inclusive examination of these data evaluating physical activity and sedentary behavior in all cancer types, of both short- (<5 years since diagnosis) and long-term (≥ 5 years since diagnosis) survivors. Previous studies have generally excluded short-term survivors on the basis that these individuals were still likely receiving cancer treatment, suggesting that the side effects of these treatments had the potential to affect activity levels [24,25]. Yet, a systematic review of physical activity interventions in cancer survivors conducted during the same time frame as the 2003–2004 and 2005–2006 NHANES series suggests that over 63% of these interventions take place during cancer treatment [3]. Further, there is consistent evidence to suggest that physical activity is safe both during and after cancer treatment [4], signifying that short term survivors may still be participating in physical activity. As such, it is imperative that this population be included in these analyses.

Further, as more information becomes available regarding the association between sedentary behavior and cancer outcomes, it is of critical importance to objectively evaluate time spent sedentary in all cancer types. For example, data suggest that sedentary behavior has been associated with colorectal cancer risk [13], yet previous analyses of this data have not included survivors of this type. Hence, the aim of this analysis is to objectively describe the physical activity and sedentary behavior of US cancer survivors of various tumor types using NHANES accelerometry data. We also explored the differences between cancer survivors and age-matched non-cancer survivors with respect to meeting physical activity guidelines and time spent in various physical activity and sedentary behavior categories.

## Materials and methods

### Study population

NHANES collects health information using a multistage probability design, resulting in a study sample that is representative of the non-institutionalized US population [29]. Data for the current study, which was conducted in 2015, were compiled from the 2003–2004 and 2005–2006 measurement cycles of NHANES, when an objective measurement of ambulatory activity substudy was added to the medical examination portion of the survey. The National Center for Health Statistics Research Ethics Review Board approval and documented consent was obtained from all participants.

### Sedentary behavior and physical activity assessment

A hip-mounted accelerometer, the Actigraph AM-7164 (ActiGraph, Fort Walton Beach, FL, USA), worn during all waking hours for one week, provided objectively-measured ambulatory activity data in the form of activity counts per minute (cpm). Intensity of activity was categorized according to the following well-established and widely used cutpoints [30]: <100cpm, sedentary behavior; 100–1,951 cpm, light-intensity physical activity; 1,952–5,724 cpm, moderate-intensity physical activity; and ≥5,725 cpm vigorous-intensity activity. These cutpoints have been used in previous examinations of NHANES cancer survivors [26–28], and proved to be better suited for the current study population than other commonly used cutpoints [22] in sensitivity analyses (results not shown). Time spent in each category was averaged across valid days to provide an average daily duration (min/day). The average daily time spent in activity of at least moderate intensity (both in total minutes and time spent in ≥10 minute bouts) was multiplied by seven to obtain a weekly average (min/wk) and used to create a dichotomous variable differentiating those with and without sufficient activity to meet the recommended amount of 150 min/wk of moderate to vigorous physical activity.

Ten hours of wear time was considered a valid day of wear. This was calculated by subtracting non-wear time (i.e. periods of at least 60 consecutive minutes of no activity with an allowance for 2 consecutive minutes of observations between 1 and 100 counts) from the total daily observation time [22]. Initially, a minimum of four valid days of monitor wear was considered for inclusion into the study sample. However, this conservative requirement and the relatively rare occurrence of certain cancer subtypes led to prohibitively small subsample sizes. Therefore, as has been done in previous studies [22,31], participants were included in the study sample with at least one valid day of wear in order to retain as many participants as possible. A sensitivity analysis was conducted comparing activity from at least one valid day of wear to four days, and no significant differences were found (results not shown).

### Cancer coding

Information on cancer status was obtained from the Medical Conditions portion of the survey questionnaire. Those who answered “yes” to whether they were “ever told you had a cancer or malignancy” (MCQ220) were considered a cancer survivor, while those who answered “no” were considered a non-cancer survivor. Cancer survivors then identified their type(s) of cancer from a list of 32 options (MCQ230). Those who identified non-melanoma skin cancer as their only type of cancer were removed from the analysis as these cancers are typically non-invasive and have a low mortality burden. The remaining cancer survivors (n = 508) were then categorized by cancer type into the following groups: breast (n = 103), cervix (n = 56), colorectal (n = 44), melanoma (n = 41), prostate (n = 93), uterine (n = 31), multiple cancers (n = 18), unknown cancer (individuals who answered “yes” to question MCQ220, but selected “cancer type unknown” from the list of options in question MCQ230; n = 30), and other cancers (which include bladder, blood, bone, brain, esophagus, gallbladder, kidney, larynx/windpipe, leukemia, liver, lung, lymphoma, mouth/tongue/lip, nervous system, ovary, pancreas, soft tissue, stomach, testis, thyroid; n = 92). Cancer survivors’ age at first diagnosis (MCQ240) was subtracted from their age at the time of the survey to determine time since diagnosis. This variable was used to dichotomize the sample into short-term (<5years, n = 168) and long-term (≥5years, n = 336) cancer survivors.

### Statistical analysis

All analyses were performed in SAS 9.3 (SAS Institute, Cary, NC) using PROC SURVEY procedures and weights provided by NHANES to account for the oversampling of certain population subgroups. Estimates of survey population means for physical activity and sedentary behavior variables were calculated by cancer status; and by cancer type, demographic variables, and time since diagnosis in cancer survivors. Differences in participant characteristics between the cancer and non-cancer groups, who were 2:1 age-matched to the cancer group within each decade of life, were analyzed using independent samples t-tests for continuous variables and chi-squared tests for categorical variables. Measures of central tendency were calculated for each of the activity variables by category of cancer type, gender, race/ethnicity, and time since diagnosis for descriptive purposes. Differences between the cancer and non-cancer group on all activity variables were analyzed using linear and logistic regression. Non-linear variables (daily and weekly moderate-to-vigorous physical activity) were log transformed and were tested as geometric means where applicable. An unadjusted model and a model adjusted for gender, race/ethnicity, marital status, current smoking status, and accelerometer wear time were fitted.

## Results

### Study population

Of the 20,470 individuals to participate in the 2003–2004 and 2005–2006 versions of NHANES, 14,616 children and adults took part in the objective measurement of ambulatory activity substudy. Of those, 12,721 participants had at least one valid day of accelerometer wear. Of those, 693 individuals self-identified as having a history of a cancer diagnosis. As only adults were queried on cancer status, the sample was delimited to those age ≥ 20 years (n = 7,564). After removing those diagnosed with non-melanoma skin cancer (n = 172), as well as those without all relevant control and predictor variables (n = 13), an analytic sample of 508 cancer survivors remained. After matching the non-cancer survivors on age, the final analytic sample yielded 508 cancer survivors and 1,016 non-cancer survivors.

Participant characteristics dichotomized by cancer status can be found in Table 1. Cancer survivors had an average age of 61.4 (95% CI: 59.6, 63.2) years; 57% were female. When compared to non-cancer survivors (average age: 62.3 (95% CI: 60.0, 62.5); 48% female), cancer survivors were more likely to be female (*P* < 0.001), non-Hispanic White (*P* < 0.001), and more likely to be current smokers (*P* < 0.05). Accelerometer wear time was also higher among non-cancer survivors as compared to survivors (20 minutes/day; *P* < 0.01).

**Table 1:** Characteristics of sample by cancer status.

	Cancer Status		
	No (n = 1,016)	Yes (n = 508)	*P*-value
***Characteristic***			
Age^a^	62.3 (60.0, 62.5)	61.4 (59.6, 63.2)	0.89
Sex (number of females)	487 (48%)	290 (57%)	<0.001
Ethnic Background			<0.001
Non-Hispanic White	575 (57%)	375 (74%)	
Non-Hispanic Black	182 (18%)	80 (16%)	
Mexican American	209 (21%)	34 (7%)	
Other	50 (5%)	19 (3%)	
Education			0.73
<High School	369 (36%)	145 (29%)	
Completed High School	246 (24%)	138 (27%)	
Some College	226 (22%)	128 (25%)	
College Grad & Above	173 (17%)	96 (19%)	
Income Level			0.79
Under $20k	303 (31%)	138 (28%)	
$20-45k	317 (33%)	162 (34%)	
$45-75k	186 (19%)	99 (21%)	
$75k+	163 (17%)	79 (17%)	
Marital Status			0.03
Married	609 (60%)	315 (62%)	
Single	280 (28%)	113 (22%)	
Divorced	127 (13%)	80 (16%)	
BMI^a^	28.8 (28.3, 29.3)	27.9 (27.3, 28.6)	0.07
Current smoker	163 (16%)	94 (19%)	<0.05
Comorbidities			
CVD (% yes)	40 (4%)	17 (3%)	0.86
DM (% yes)	165 (16%)	72 (14%)	0.25
Accelerometer Wear Time			
Total (min/day)^a^	862 (851, 873)	842 (828, 855)	<0.01
% Time Worn at Each Intensity			
LPA	37%	36%	0.24
MVPA	2%	2%	0.07
Sedentary Behavior	61%	62%	0.15

^a^ Values are population weighted means (95% CI). Categorical variables compared between categories

by chi-square, continuous variables compared using t-test.

^¥^ Participants missing from analysis

Education (n = 3); Income (n = 77); DM (n = 4); BMI (n = 13).

### Physical activity & sedentary behavior

Cancer survivors spent 36% of waking time engaged in light-intensity physical activity, 2% in moderate-to-vigorous intensity physical activity, and 62% in sedentary behavior. On average (unadjusted means), cancer survivors engaged in 307 (95% CI: 295, 319), 15 (95% CI: 13, 17), and 0 (95% CI: 0, 1) min/day of light-intensity physical activity, moderate-intensity physical activity, and vigorous-intensity physical activity, respectively, with 8% sufficiently meeting the physical activity recommendations. These individuals also reported a total of 519 (95% CI: 506, 532) minutes of sedentary time, with 86 (95% CI: 84, 88) breaks in sedentary behavior per day. Full descriptive results of physical activity and sedentary behavior stratified by cancer type and demographic characteristics are presented in Table 2. There was insufficient statistical power to fully test for differences in physical activity and sedentary behavior among cancer survivors with respect to all demographic characteristics. For exploratory purposes, we dichotomized the sample with respect to the following key variables: age (<60, n = 148 *vs*. ≥60, n = 360), race/ethnicity (White, n = 375 *vs*. Other, n = 133), gender, and time since diagnosis. There were significant differences in the age and gender subgroups, with older adults performing significantly less light- (85 minutes/day; *P* <0.01), moderate-vigorous (12 minutes/day; *P* <0.01), and total moderate-to-vigorous-intensity physical activity per week (82 minutes/week; *P* <0.01). These individuals also spent an average of 95 minutes more per day sedentary (*P* <0.01) with 11 fewer breaks in sedentary time (*P* <0.01) than younger adults. With respect to gender, we found that females performed 30 more minutes of light-intensity physical activity per day (*P* <0.01), as well as 54 fewer minutes sedentary (*P* <0.01) and four more breaks in sedentary behavior (*P* = 0.01) than their male counterparts. There were no significant differences in any activity category by race or time since diagnosis.

**Table 2:** Physical activity and sedentary behavior of cancer survivors by cancer type & demographic characteristics.

		Intensity-specific categories of physical activity (minutes per day)				Weekly moderate-to-vigorous intensity physical activity (MVPA)		
	N	Light	Moderate-Vigorous	Sedentary	Sedentary breaks (breaks/day)	Total accumulated MVPA (min/wk)	MVPA within bouts of ≥10 min (min/wk)	Meeting guidelines ^b^ (%)
**Cancer Type**		Mean (95% CI)	Mean (95% CI)	Mean (95% CI)	Mean (95% CI)	Mean (95% CI)	Mean (95% CI)	
Breast	103	295 (273, 316)	14 (9, 19)	525 (497, 553)	84 (81, 88)	95 (60, 130)	34 (17,50)	11%
Cervix	56	366 (328, 404)	18 (14, 23)	442 (416, 469)	91 (87, 95)	129 (96, 161)	26 (9, 42)	2%
Colorectal	44	278 (246, 309)	9 (6, 12)	576 (535, 617)	83 (77, 89)	64 (43, 87)	18 (3, 34)	2%
Melanoma	41	298 (269, 327)	18 (11, 25)	520 (477, 563)	82 (77, 87)	126 (76, 177)	33 (13, 53)	5%
Prostate	93	259 (237, 281)	14 (10, 18)	566 (543, 589)	78 (74, 83)	96 (67, 125)	43 (20, 65)	13%
Uterine	31	314 (263, 365)	14 (9, 19)	521 (465, 577)	93 (83, 103)	97 (61, 134)	37 (15, 58)	12%
Multiple	18	317 (250, 384)	12 (4, 20)	528 (473, 583)	92 (79, 105)	84 (28, 140)	14 (0, 28)	0%
Unknown	30	323 (269, 377)	17 (8, 27)	517 (471, 562)	90 (81, 99)	120 (55, 185)	28 (6, 51)	4%
Other	92	309 (284, 335)	17 (12, 23)	525 (495, 554)	87 (83, 91)	121 (83, 160)	38 (15, 61)	10%
**Demographic characteristics**								
Age		Mean (95% CI)	Mean (95% CI)	Mean (95% CI)	Mean (95% CI)	Mean (95% CI)	Mean (95% CI)	
<60	148	353 (339, 366)	22 (18, 25)	468 (451, 485)	92 (89, 94)	153 (127, 178)	39 (26, 52)	9%
≥60	360	268 (257, 280)	10 (9, 12)	563 (545, 580)	81 (79, 83)	71 (61, 82)	29 (22, 35)	7%
	*P*-value ^a^	<0.001	<0.001	<0.001	<0.001	<0.001	0.01	0.81
Gender								
Male	218	287 (269, 305)	18 (15, 21)	555 (537, 573)	83 (79, 87)	127 (104, 150)	42 (30, 54)	11%
Female	290	317 (302, 332)	14 (12, 16)	501 (483, 519)	87 (85, 90)	99 (85, 114)	29 (21, 36)	7%
	*P*-value ^a^	<0.001	0.24	<0.01	0.01	0.24	0.44	0.24
Race/Ethnicity								
White	375	308 (294, 321)	16 (14, 18)	518 (503, 532)	85 (83, 88)	113 (100, 127)	35 (29, 42)	9%
Other	133	304 (281, 327)	11 (8, 15)	529 (504, 555)	89 (85, 93)	80 (57, 103)	18 (7, 29)	4%
	*P*-value ^a^	0.09	0.97	0.13	0.30	0.97	0.59	0.42
Time since Dx^c^								
<5 years	168	296 (277, 315)	16 (12, 19)	534 (510, 558)	85 (81, 89)	111 (87, 135)	39 (21, 58)	11%
≥5 years	336	312 (297, 326)	15 (13, 17)	513 (497, 528)	86 (84, 89)	107 (90, 123)	29 (22, 36)	7%
	*P*-value ^a^	0.09	0.97	0.13	0.30	0.97	0.59	0.42

P values correspond to the difference between dichotomized variables (includes all cancer types)

^a^ Adjusted for wear time

^b^ At least 150 min/week spent in bouts of MVPA lasting ≥10 min, using Freedson cutpoints [30]

^c^ Four participants either did not report or did not know age at diagnosis

Non-cancer survivors spent an average (unadjusted means) of 319 (95% CI: 313, 326), 18 (95% CI: 16, 20), and 1 (95% CI: 0, 1) min/day in light-intensity physical activity, moderate-intensity physical activity, and vigorous-intensity physical activity, respectively. Additionally, 8% of these individuals met physical activity guidelines. This group spent an average of 524 (95% CI: 514, 534) minutes per day sedentary, with an average of 87 (95% CI: 86, 89) breaks in sedentary time per day. In regard to our secondary aim, which was to evaluate differences in activity patterns between cancer survivors and non-cancer survivors, we found odds ratios of 0.97 (0.60, 1.58) and 1.10 (0.67, 1.80) of meeting physical activity guidelines, respectively (Table 3). After adjusting for gender, race/ethnicity, marital status, current smoking status, and accelerometer wear time, cancer survivors performed significantly less light-intensity physical activity (*P* <0.01) and engaged in more sedentary time (*P* <0.01) with fewer breaks in prolonged sitting (*P* = 0.04).

**Table 3:** Physical activity and sedentary behavior by cancer status.

		Intensity-specific categories of physical activity (minutes per day)				Weekly moderate-to-vigorous intensity physical activity (MVPA)		
Cancer Status		Light	Moderate-Vigorous	Sedentary	Sedentary breaks (breaks/day)	Total accumulated MVPA (min/wk)	MVPA within bouts of ≥10 min (min/wk)	Meeting guidelines^b^ (%)
	N	Mean (95% CI)	Mean (95% CI)	Mean (95% CI)	Mean (95% CI)	Mean (95% CI)	Mean (95% CI)	(%)
Not Diagnosed with Cancer	1016	319 (313, 326)	18 (16, 20)	524 (514, 534)	87 (86, 89)	128 (115, 142)	40 (33, 47)	8%
Cancer Survivors (All Types)	508	307 (295, 319)	16 (14,17)	519 (506, 532)	86 (84, 88)	109 (96, 121)	33 (27, 40)	8%
Unadjusted difference^c^		12 (2, 22)	1 (1, 1)	5 (-8, 18)	2 (-1,4)	1 (1, 1)	1 (1, 2)	OR* = 0.97 (0.60, 1.58)
*P*-value		0.02	0.16	0.48	0.13	0.16	0.17	
Adjusted^a^ difference^c^		12 (3, 21)	1 (1, 1)	-13 (-24, -3)	2 (0, 3)	1 (1, 1)	1 (1, 2)	OR = 1.10 (0.67, 1.80)
*P*-value		0.01	0.53	0.01	0.04	0.53	0.50	

^a^Adjusted for gender, race/ethnicity, marital status, current smoking status, and accelerometer wear time.

^b^ At least 150 min/week spent in bouts of MVPA lasting ≥10 min, using Freedson cutpoints[30]

^c^ Least squares means and 95% CI (except for “Meeting Guidelines” where odds ratio and 95% CI for meeting guidelines between non-cancer vs cancer survivors.

*As compared to the reference group (Cancer Survivors).

## Discussion

The use of accelerometry in estimating physical activity and sedentary behavior at the population level has been a critical development in our fundamental understanding of these behaviors. In order to understand the scope of the problem and optimize the efficacy of interventions to promote physical activity and reduce sedentary behavior in cancer survivors, we must understand the prevalence of activity within each cancer type, as well as how this relates to activity levels in the general population. This study used data from the 2003–2004 and 2005–2006 versions of NHANES to provide insight into the amount of physical activity and sedentary behavior performed among cancer survivors. To our knowledge, this study is the first to analyze both physical activity and sedentary behavior utilizing objective measures in a nationally representative sample of short- and long-term cancer survivors of all types. As discussed previously, Troiano, et al. [22] found that when using these data, the percentage of U.S. adults meeting physical activity recommendations was actually quite low, especially when compared to studies using self-report. Accordingly, our analysis found that only 8% of our sample of adult cancer survivors were meeting physical activity guidelines. This is very similar to the percentage (7%) found by Trioano, et al. [22]. Interestingly, we did not find a significant difference between the percentage of cancer survivors and age-matched non-cancer survivors (8%) meeting these guidelines. Previous analyses using 2003–2006 NHANES data found slightly different percentages of cancer survivors meeting guidelines (12% [24]; 5% [25]), though this can likely be explained by the exclusion of both certain cancer types [24] and short-term survivors [24,25]. Ultimately, when collectively viewed with previous research, our study continues to suggest that the vast majority of cancer survivors, like the general population, do not participate in sufficient amounts of physical activity.

Previous studies describing physical activity using NHANES accelerometer data in cancer survivors have focused on specific cancer types (breast, prostate, colon, and endometrium) [24,26–28] and long-term survivors [24,25]. Our findings mirror these studies in that we found that very few survivors, including cervical and melanoma cancers, as well as those with multiple cancers, are participating in moderate-to-vigorous intensity physical activity. In fact, the vast majority of this sample is performing none at all. While we were not powered to statistically test for differences in meeting the guidelines between cancer types, it does appear as though survivors of some cancer types (cervical, colorectal, melanoma, and those with multiple cancers) are meeting the guidelines less frequently than others. When looking specifically at light-intensity physical activity, the average time spent in light-intensity physical activity is almost identical to the above studies, suggesting that most of cancer survivors’ activity takes place at this level of intensity. This is noteworthy in that, while there are many documented benefits of moderate-to-vigorous-intensity physical activity [4], for some groups of survivors, interventions that focus on the type of activity that they actually do (*i*.*e*., light intensity) may be more feasible and appealing. As such, this information can be useful in developing successful future interventions in this population.

Importantly, in this first analysis to incorporate short-term cancer survivors, we found no significant differences in any activity or sedentary category between long-term and short-term cancer survivors. Previous studies of physical activity and cancer survivorship have generally excluded short-term survivors on the basis that these individuals were still likely receiving treatment for their cancer and could potentially still be suffering lingering side effects of treatment that could influence their activity levels [24,25]. Our study suggests that short-term survivors appear to exhibit similar activity and sedentary behavior patterns as long-term survivors. One possible implication of this finding is that short-term cancer survivors should be included in future study at the population level, as well as in physical activity and sedentary behavior interventions.

With respect to the amount of time spent sedentary, previous analyses of these NHANES data have only described these behaviors in breast [26,27] and prostate [28] cancer survivors. With the inclusion of many cancer types in our analysis, we observed that, on average among all survivors, the amount of time spent in sedentary behavior is similar to that of breast and prostate cancer survivors. This information is important in that it provides an estimate of the sedentary behavior of survivors of common cancer types that have previously been linked with sedentary behavior. For instance, increased sedentary behavior has been associated with colorectal cancer risk [13,14], the third most common cancer and leading cause of cancer death for both men and women [32]. While we were underpowered to evaluate differences in sedentary behavior between cancer types, there appears to be potential differences in sedentary behavior between certain cancer types. As such, it is imperative that we continue to examine sedentary behavior objectively at the population level to truly describe these behaviors among survivors.

Our analysis, the first study to explore differences in objectively measured sedentary behavior in cancer survivors vs. non-cancer survivors using the NHANES data, did suggest that cancer survivors are more sedentary than their non-cancer survivor counterparts, suggesting that interventions specifically aimed at reducing and re-patterning (breaking up) sedentary behavior are needed in this population. While an optimal dose of sedentary reduction has yet to be determined, previous analyses of cancer survivors suggest that greater time spent sedentary was found to be positively associated with an increased risk for all-cause mortality and cancer specific-mortality [33], as well an increased BMI, diminished quality of life, and possible risk of cancer progression in cancer survivors [14]. Within cancer survivors, we found that older adults tended to be more sedentary (with fewer breaks) than younger adults. Older cancer survivors in particular may be an attractive target for these interventions, as older adults spend up to 70% of their waking hours sedentary [33] and may be more likely to avoid traditional exercise. This study was also the first to use NHANES data to evaluate breaks in sedentary time among cancer survivors. Sedentary breaks haven been associated with improved waist circumference, BMI, triglycerides, and 2-hr plasma glucose in the general population [17,34]. Again, we found that cancer survivors take significantly fewer breaks in sedentary time than do non-cancer survivors, highlighting the need for future interventions in these survivors.

There are many strengths to this study, including the large, nationally-represented sample of cancer survivors of multiple cancer types. Strong measurement methods were used; the ActiGraph is a reliable and valid of physical activity and sedentary behavior [20] that limits the error and inherent biases often associated with subjective measures. Further, accelerometers do a better job of capturing light-intensity physical activity and sedentary behavior than do self-report [17,19]. The ability to estimate light-intensity activity accurately is especially important in this population, in that, as demonstrated by these data, this population tends to perform more light-intensity physical activity than moderate or vigorous intensity physical activity.

This study also had several limitations. First, although we had a large sample of cancer survivors, the small number of survivors within strata did not allow us to test for differences between cancer types and all demographic characteristics. The cross-sectional nature of this study also did not allow us to evaluate temporal differences in physical activity and sedentary behavior, nor does it allow us make conclusions regarding the directionality of the relationship between cancer and activity patterns. While the use of a hip-mounted accelerometer has improved the estimation of physical activity and sedentary behavior, there are inherent limitations to its use. Some physical activity is unsuccessfully captured by the accelerometer, such as cycling or swimming. Further, our choice of acceleration cut-points [35] to describe these data by intensity level may lead to potential misclassification of intensity in certain activities. Accelerometry also allows for potential measurement error in the approximation of sedentary behavior due to its inability to differentiate between sitting and standing. However, work comparing accelerometers to direct observation and to the activPAL, a device with an inclinometer, which can detect changes in posture, suggest this error to be less than 5% [36]. Still, as there may be significant health benefits to decreasing sitting time by increasing standing, this delineation is an important one. Future population studies specifically evaluating sedentary behavior should attempt to include devices with an inclinometer. Finally, it should be noted that these data was collected over ten years ago. As such, there is a possibility that they may not fully represent the current physical activity and sedentary behavior patterns of cancer survivors.

In summary, the use of objective measures of physical activity and sedentary behavior to more accurately describe these behaviors in a population is instrumental in our understanding of cancer survivors. These findings suggest that, like the general population, the vast majority of cancer survivors are not meeting physical activity guidelines. This appears to be consistent among both short-term and long-term survivors. Further, cancer survivors are significantly more sedentary, while taking fewer breaks in their sedentary time, than their non-cancer survivor counterparts. The amount of time these survivors are sedentary reflect previous findings specific to breast and prostate cancer survivors. As this population may greatly benefit from physical activity and sedentary behavior as a therapeutic modality, future work is needed to test interventions along the cancer continuum.
